# Microtechnologies for Cell Microenvironment Control and Monitoring

**DOI:** 10.3390/mi8060166

**Published:** 2017-05-23

**Authors:** Enrique Azuaje-Hualde, Maite García-Hernando, Jaione Etxebarria-Elezgarai, Marian M. De Pancorbo, Fernando Benito-Lopez, Lourdes Basabe-Desmonts

**Affiliations:** 1 BIOMICs Microfluidics Research Group, Microfluidics Cluster UPV/EHU, Lascaray Research Center, University of the Basque Country UPV/EHU, 01006 Vitoria-Gasteiz, Spain; enrique.azuaje@ehu.eus (E.A.-H.); maite.garciah@ehu.eus (M.G.-H.); jaione.etxebarria@ehu.eus (J.E.-E.); 2 Analytical Microsystems & Materials for Lab-on-a-Chip (AMMa-LOAC) Group, Microfluidics Cluster UPV/EHU, Analytical Chemistry Department, University of the Basque Country UPV/EHU, 01006 Vitoria-Gasteiz, Spain; fernando.benito@ehu.eus; 3 BIOMICs Research Group, Lascaray Research Center, University of the Basque Country UPV/EHU, 01006 Vitoria-Gasteiz, Spain; marianpancorbo@gmail.com; 4 IKERBASQUE, Basque Foundation of Science, 48011 Bilbao, Spain

**Keywords:** cell analysis, high-throughput, microfluidics, microtechnology

## Abstract

A great breadth of questions remains in cellular biology. Some questions cannot be answered using traditional analytical techniques and so demand the development of new tools for research. In the near future, the development of highly integrated microfluidic analytical platforms will enable the acquisition of unknown biological data. These microfluidic systems must allow cell culture under controlled microenvironment and high throughput analysis. For this purpose, the integration of a variable number of newly developed micro- and nano-technologies, which enable control of topography and surface chemistry, soluble factors, mechanical forces and cell–cell contacts, as well as technology for monitoring cell phenotype and genotype with high spatial and temporal resolution will be necessary. These multifunctional devices must be accompanied by appropriate data analysis and management of the expected large datasets generated. The knowledge gained with these platforms has the potential to improve predictive models of the behavior of cells, impacting directly in better therapies for disease treatment. In this review, we give an overview of the microtechnology toolbox available for the design of high throughput microfluidic platforms for cell analysis. We discuss current microtechnologies for cell microenvironment control, different methodologies to create large arrays of cellular systems and finally techniques for monitoring cells in microfluidic devices.

## 1. Introduction

**Figure 1:**
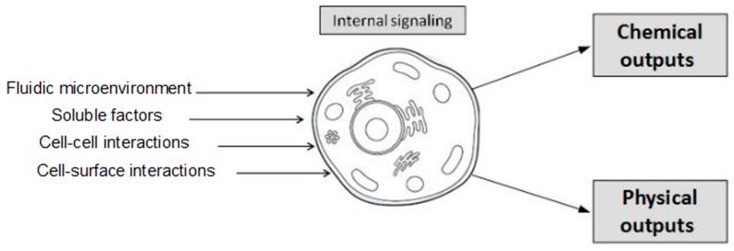
Input signals from cell microenvironment induce internal signaling of cells and modulate their outputs, affecting cell behavior.

Native cells are in a dynamic multifactorial environment, their own microenvironment. The cell microenvironment is constituted by: their extracellular matrix (ECM), the topography and physical properties of the ECM and by soluble factors on their fluidic environment. All of them strongly affect cell fate and cell behavior. Changes in the cell microenvironment are transduced into intracellular signaling pathways, which regulate cell fate and cell behavior. Conventional cell culture systems often rely on batch experiments with limited control of cell microenvironments. In order to obtain a comprehensive knowledge of cell function and behavior, it would be desirable to develop experimental methods that could explain the contribution of each of those environmental factors, as well as their synergetic effects on cell behavior (Figure 1).

During the last two decades, we have witnessed a number of key developments in the area of the microtechnologies, which allows introducing control and complexity over a full range of environmental factor at the microscale level. For example, technologies for the accurate structuration of surfaces for subsequent cell culture, microfluidic architectures, synthesis of novel biomaterials and nanomaterials with sensing and actuating capabilities have been developed and their potential for cell culture, stimulation and analysis has been proven. In particular, the miniaturized scale of microchannels in microfluidic devices offers advantages such as low contamination risk, fast transfer of nutrients and heat, short equilibration times, parallelization of processes and automation, low reagent and power consumption, portability, etc. Moreover, since the dimensional environment is analogous to in vivo conditions, the small sizes of the channels permit medium and nutrients to diffuse to nutrient-poor areas. Currently, there is little development of microtechnologies that can successfully mimic the in vivo microenvironments, since any change in material, surface chemistry, cell number or flow conditions can affect the results of the assays [1]. Nowadays, there is an increasing use of microfluidic techniques on cell culture that have opened a broad range of possibilities for studying cells in a variety of contexts, allowing to understand the specific contribution of each different parameter to cellular behavior, such as shear forces, nutrient gradients, etc. [2]. An extra advantage of the use of microtechnologies is the scalability and the possibility of parallelization of cellular samples which allow high-throughput (HTP) measurements, essential for the statistical analysis of multi-parameter environments, and for the construction of predictive models.

The current trend is to develop HTP and multiplexed technologies, essentially those who also allow a real time or near-real time analysis for both single cell and multi cell platforms. The properties that can be quantified from analysis includes the study of the cells mechanics (deformation, migration and growth), the proteome, genome and secretome, and both their extracellular and intracellular interactions and their stimuli [3].

Integration of several microtechnologies to create controlled multi-parametric environments and monitoring is still a challenge. Microfluidics has emerged as a new way to fabricate large cellular arrays in defined patterns which allows the study of a large number of cells in a specific microenvironment as well as the observation and quantification of several outcomes from a single study. Looking for the best way to design novel platforms for cell analysis, in this manuscript, we review examples on how different parameters of cell microenvironment may be controlled through microtechnologies, as well as the techniques available for monitoring cells in microfluidic devices, centering on the analysis of chemicals outcomes. Additionally, we give an overview of current microfluidic platforms already available for cell analysis.

## 2. Microtechnologies for Cell Microenvironment Control

**Figure 2:**
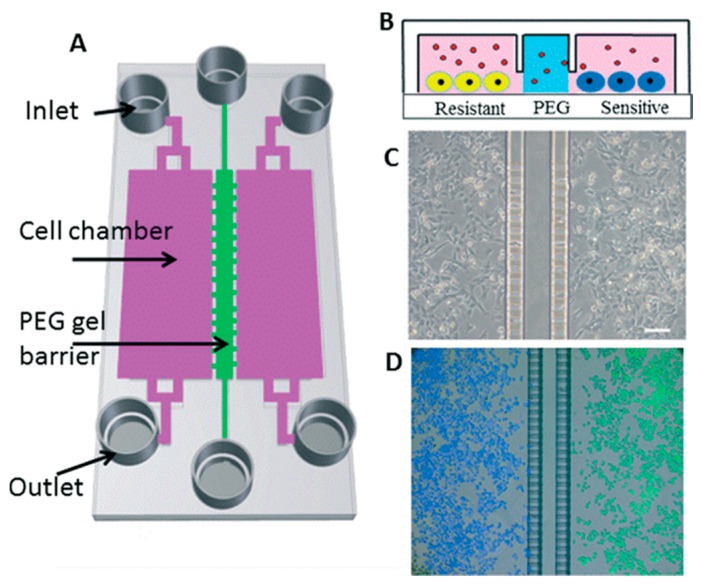
An example of a microfluidic co-culture device with semi-permeable PEG barrier to control soluble factors in small volumes. (**A**) Schematic of a device with two 1.8 mm-wide cell culture chambers in pink separated by a 100 μm PEG gel channel in green. (**B**) Cross-sectional view of two types of melanoma cells cultured in close proximity. (**C**) Brightfield image of A-375 cells cultured at Day 1. Scale bar represents 100 μm. (**D**) A-375 cells were labeled with either green or blue cell-tracker dyers before being seeded in alternate chambers. Migration was assessed after a 48 h culture period. Reproduced from [8] with permission of The Royal Society of Chemistry.

The term “cell microenvironment” involves those factors that directly or indirectly affect cell behavior, by biophysical, biochemical or other pathways. As previously stated, those factors can be classified in: ECM, cells surrounding a single cell, soluble factors, topography or physical properties of the ECM, and fluidic cues. All of them strongly affect cell proliferation and differentiation. However, despite this distinction, each factor cannot be considered independently, as all affect cell behavior in an orchestrated manner. During the past few years, the development of microfluidic platforms has been focused on the production of an optimal microenvironment that replicates cells natural conditions and allows their manipulation, Figure 2. The use of microfluidics brings many advantages in regulating cellular microenvironment, since microscale technologies make possible the control of soluble factors, cell adhesion to the surface, and also cell–cell contact [4].

To study cellular behavior in similar conditions to the in vivo responses, two approaches can be taken: study the interaction of each cell with their respective microenvironment or study the general interaction between the cell microenvironment and a cluster of cells. The isolation of cells allows the study of cell population heterogeneity, which enables analyzing the responses and behavior of a large number of individual cells and therefore distinguishing between cell subpopulation [5]. On the contrary, microfluidic multi-cell studies offer information about the entire cell population. These types of cultures have been largely used to try to understand the general responses of an entire cellular population to a specific reagent [6,7]. By housing a small number of cells in microwells or spots, both the cell–cell interactions and microenvironment changes can be observed and quantified, opposite to the single cell studies. In general, to carry out either of these studies, it is necessary to have under control the specific microenvironmental parameters.

### 2.1. Topography

**Figure 3:**
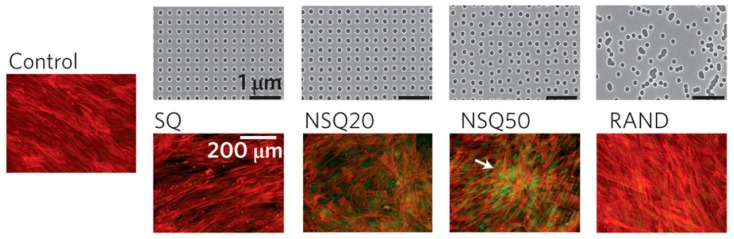
Topographical manipulation by electron-beam lithography. Osteoinduction (Generation of bone tissue) of MSCs was not obtained neither in square (SQ) nor random (RAND) patterns, while appearing on the controlled disorder patterns (NSQ20 and NSQ50 same as SQ but with ±20 nm and ±50 nm offset from the 300 nm center–center position) Adapted from [10] with the permission of Nature Publishing Group.

Cells are capable of sensing and responding to different signals, including those provided by the ECM. In vivo, ECM has a specific physical structure and molecular composition; it presents a variety of geometrically-defined three-dimensional (3D) physical cues on the order of micron and sub-micron scale known as topographies. The interaction between cells and topography is driven by a phenomenon named “contact guide”, and it is related to cell adhesion, morphology and differentiation [9]. Therefore, being capable of modifying surfaces at nano- and micro-scale emerges as crucial to study cell–surface interactions, as shown in Figure 3, where mesenchymal stem cells (MSCs) differentiation to bone tissue is affected by the topography of the substrate.

One of the main interests in topography in the last years lies on its effect on Mesenchymal Stem Cells (MSC), where changes on surface properties, proliferation and differentiation can be caused by cell–ECM or cell–surface interactions. Recently, it has been confirmed that, when mouse Embryonic Stem Cells (mESC) are cultured on poly(2-hydroxyethyl methacrylate-*co*-ethylene dimethacrylate) **(**HEMA-EDMA) surfaces with hierarchical micro to nano roughness, their long-term self renewal is promoted, while, when culturing them in contact with smooth or nanorough polymer surfaces, differentiation is induced [11]. As reported by Jaggy et al., when cells were cultured on controlled topographies, hierarchical micro-nanoscale surface roughness favored the pluripotent character of mESCs when compared to a chemically identical nano rough or smooth surface. Because of the previous experiments, roughness was identified to maintain stem cell self-renewal capabilities, as long as the surface chemistry remained hydrophilic.

There is a wide spectrum of biocompatible techniques available to perform topographical features at microscale. It is worth mentioning that biocompatibility of the fabrication techniques and the materials used is a crucial factor in ensuring cell viability on those rough surfaces incorporating micro/nanofabricated structures [12,13].

Until now, photolithography is the most extended technique to make micro- and nanostructures in flat surfaces, while other biocompatible microfabrication techniques based on 3D printing, electrospinning and self-assembly are currently emerging [14]. Currently, soft lithography is the most used technique, however, other lithography methods are being developed in order to overcome the drawbacks it still has, such as the need of knowledge on Microcontact Printing process (µCP), or the difficulties to create multiple protein patterns easier.

Actually, various researches focused on the studying of migration in different topographical microenvironments [15,16]. As an example, a promising method was developed by Kon and co-workers, based on a protein-friendly photoresist, combining Capillary Force Lithography (CFL) and Microscope Projection Photolithography (MPP), to study cellular dynamics under topographical microenvironments [17]. This lithography method was adequate to create multiscale multiple protein patterns, even though it did not reach good precision in the creation of submicrometer features. Nevertheless, this technique was meaningful to study the effect of topography on human colon cancer cells, demonstrating that hierarchically organized structures on the surface, modulates processes such as cell migration and adherence.

In contrast, when it comes to high precision, Nanoimprint Lithography (NIL) emerges as a technique capable of creating nanostructures with molecular resolution [18]. NIL and surface functionalization have been used to create RGD peptide functionalized nanodots on silicon surfaces. As cells tend to adhere to RGD peptide, the combination of these two techniques has been successfully used to study of the effect of surface nanostructures on MSC behavior.

Furthermore, a method based on “multiphoton ablation lithography” has been developed to create patterns of different pitches and constant nanocrater dimensions on a surface. Topographical structures were produced by “multiphoton ablation lithography”. It has been demonstrated that modifying surfaces with topographical structures at the nano-scale, cell behavior without any chemical treatment of the substrate can be modulated, showing a clear trend of the cells to certain features [16].

Nanoparticles have also been employed to modify the topography of cell culture substrates. Recently, a method called Printing and Vacuum Lithography (PnV lithography) has emerged from our laboratories, enabling multi-patterning of biomolecules and gold nanoparticles (GNPs) by combination of vacuum soft-lithography and microcontact printing. Control of the surface coatings of the nanoparticles enabled controlled NP assembly and therefore substrate topographies. This technique allowed the controlled assembly of cells in adjacent positions to multiple topographies [19].

Furthermore, a method for dynamic cell micropatterning on nanostructured surfaces was developed with a cell-friendly photoresist [20]. A device with different topographies was fabricated, and it showed how cells respond to distinct topographies during spreading. Moreover, with this device, it was possible to observe the dynamic behavior of ~30 cells simultaneously and, thus, systematically compare the influence of topography in their spreading. This technique looks promising due to its dynamic working, which proposes information read out in real time, giving a more realistic point of view of cell behavior.

### 2.2. Biochemical Factors: Surface Chemistry and Soluble Factors

**Figure 4:**
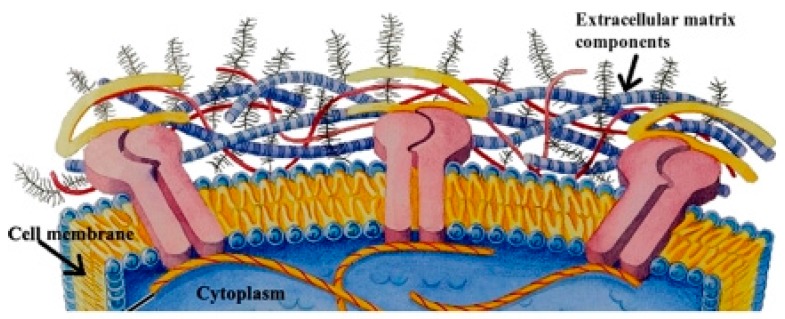
This figure shows the intimate contact between cellular membrane and the extracellular matrix (ECM, protein fibers embedded into a polysaccharide gel). Understanding the interaction between cells and ECM is essential for understanding cellular behavior. Adapted from [24] with permission from PLOS computational biology.

Cells not only interact with their bottom substrate, as they create 3D interactions with all surrounding elements. As previously stated, the cell microenvironment and its ECM composition heavily influence the cells growth, proliferation, orientation, organelles distribution and signal transduction. Thus, understanding the chemical composition of the microenvironment and the mechanism that affects the cell behavior both physically and chemically is essential to develop an artificial ECM for efficient cell studies [21]. ECMs are made of different types of macromolecules, mainly collagens, elastin, fibronectin, glycoproteins, proteoglycans and glycosaminoglycans, whose composition and structure varies from tissue to tissue. These compounds reorder, forming three-dimensional matrices; interstitial matrices surrounding cells; and pericellular matrices in direct contact with the cells [22,23] (Figure 4).

The formation and dynamics of cell adhesion mostly rely on the direct interactions made by focal adhesions (FAs). In the eukaryotic cells, integrin and integrin-like substances form transmembrane heterodimers in the cell membrane that join to the ECM, providing links between the ECM and both cytoplasmatic proteins and the actin cytoskeleton. This connection not only serves as a mechanical link, but also acts as a mechanoreceptor, regulating the cell phenotype [25,26,27].

Apart from direct interactions between the cells and the ECM, surrounding fluids play an important role in cell behavior. It is necessary for the surrounding fluids to have fluidic mechanics, which could provide a suitable chemical surrounding of nutrients and oxygen for cell growth. Soluble factor signaling is involved in regulating many biological events such as embryonic stem cell pluripotency, mammalian embryogenesis, tumor formation and metastasis. They are especially important in Stem Cell cultures, since Stem cell fate in these niches is regulated by chemical and biological microenvironments [28,29].

Nowadays, the aim is to recreate and engineer nanoscaled ECMs for the design of cell culture platforms. In order to control cell cultures and regulate their signaling pathways, synthetic platforms made by either natural or artificial biomaterials that mimic the ECM are being developed. In the past few years, this has been mostly achieved by the use of micro- and nanopatterning, in which a desired pattern of 2D or 3D motifs with the ECM compounds is reproduced in a two-dimensional or three-dimensional substrate. Microscale technologies, in the form of microfluidic platforms appear as efficient techniques to achieve a precise control of soluble factors in small volumes, and so, modulation of signals between cells would be possible [8].

Two different approaches may be taken in order to recreate ECMs, a top down one in which the ECM are reproduced first from a macroscopic level to be later applied in its entirety on the substrate, and a bottom up approach in which the ECM is created by the assembly and gathering of smaller parts over the substrate [30,31].

Three major methods have been described for the patterning of ECM compounds. The first one was a direct method in which the final composition of the matrix is directly added to the substrate [32]. The second consists of a self-assembly method, in which small components interact with each other forming a larger structure with high hierarchy [33]. Deeg et al. obtained a simultaneous control of the ligand spacing and ligand density on a 2D cell culture by a self-assembly micropatterning [34]. The third is a replication method, in which a master, previously made by lithography methods such as nanoimprint lithography or soft lithography, is used several times in order to reproduce a specific pattern [18], enabling serial repetition of experiments using a previously designed and well organized patterns. Tan et al. used this replication method for the printing of a monolayer of initiator molecules to control the polymerization process, enabling controlled cell adhesion to the designed pattern [35]. As opposed to the direct method, the self-assembly method and the replication method allows a proper reconstruction of the ECM by a nano- and micro-scaled fabrication of the ECM. The replication method also gives the opportunity of serialization of cell arrays as the master can be used multiple times.

### 2.3. Cell–Cell Contact

Direct cell–cell contact is a type of communication between cells that relies on the interaction between cells themselves and their surrounding environment that acts as both physical anchor that constrain cells and plays as signaling interchange between cells. Together with cell shape, cell–cell contact is described as an influential factor affecting cell fate decisions, and, therefore, modulating cell behavior. Moreover it is an essential parameter to be regulated in order to achieve a successful cell culture. There is a huge range of methods which enable the precise control of the contact between cells. They can be divided into two main types: techniques involving cell adhesion to the surface, and techniques based on the confinement of cells in a definite space.

**Figure 5:**
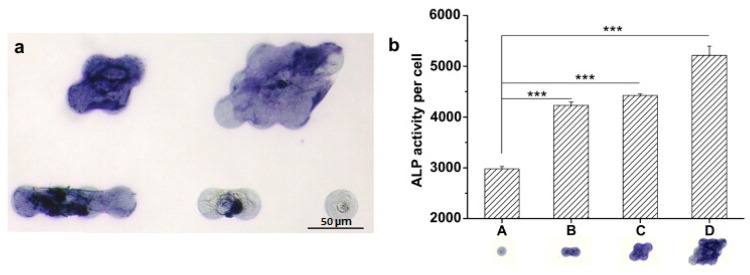
Cell–cell contacts enhance osteogenic differentiations of mesenchymal stem cells confirmed by the increased expression of alkaline phosphatase (ALP). (**a**) A brightfield images of MSCs on adhesive microdomains after six days in osteogenic differentiation medium. ALP was stained in blue by Fast Blue. Scale bar, 50 μm. (**b**) ALP activity per cell on indicated microdomains. Adapted from [37] with the permission of Elsevier.

Microtechnologies are considered powerful tools to control cell–cell interactions, leading to a certain control of cell behavior could be possible. Direct contact between cells is known to induce cell differentiation in conventional cell cultures [36]. Tang et al. confirmed that the differentiation of mesenchymal stem cells to osteogenic and adipogenic cells was regulated by cell–cell contact by using a polyethylene glycol micropatterns for controlled cell adhesion [37] (Figure 5).

In contrast, methods lacking cell adhesion have also been proven to be useful to control cell–cell contact. Interactions between cells play an essential role in creating immunologic functional cells, and therefore, in immunological response. Juxtacrine interactions or interactions that require close contact are the most important ones mediating in immunological responses. Therefore, the study of the interactions between immune cells is a good proof of principle of the capability of the microtechnologies to control cell–cell interactions. In this regard, a method based on surface acoustic valves was reported, which was capable to locate individual cells with micrometer precision, and thus enabling the control of cell–cell interactions in T-cell cultures [38]. In this work, Guo et al. demonstrated that the use of an acoustic well is useful for intercellular communication studies since the technique enables the control of the intercellular distance, the engineering of homotypic or heterotypic cell assemblies and monitoring the transformation of cellular aggregates from suspended to adherent states and the subsequent investigation of the assembly and the communication in those adherent cells. Further, in terms of real time and high throughput analysis, a method based on ultrasonic waves was developed for time-controlled induction of cell–cell contact [39]. This method allowed simultaneous trapping and aggregation of cells using ultrasonic standing waves, without affecting cell viability. Ultrasound forces were proven useful for generating cell–cell contacts, inducing synchronized contacts between Natural Killer (NK) immunological cells and giving real time information of NK cell heterogeneity.

Going to 3D, direct contact between cells is also being studied. Microfabrication techniques such as bottom-up and directed assembly of microscale hydrogels are becoming very popular, as they are considered powerful tools that enable the fabrication of 3D structures, replicating in vitro the microenvironment found in vivo. The engineering of tissues and organs, requires a scaffold which mimic cell ECM and enables cell adhesion to the surface and cell–cell contact, while it also allows the necessary soluble factors diffusion in order to get a functional organ or tissue. To mimic ECM, first, hydrogel stiffness can be controlled, in order to get the desired transport of solutes and gases through the scaffold. Moreover, hydrogel surface can be functionalized to promote cell adhesion. Finally, microtechnologies applied to produce microengineered hydrogels, allow the creation of 3D structures (scaffolds), where it is possible to precisely control the size of cell aggregates, and so, the interactions between cells [40], being capable to create a biomimetic microenvironment for cell growth. Due to the mimicking that can be achieved with microengineered hydrogels, multiple researches will be doing at this field in the near future, bringing us closer to personalized therapies.

As an example of hydrogel microfabrication, a laser printing of three-dimensional multicellular arrays method was developed to study of complex and dynamic relationships between cells and their local environment. The scaffold structure was built layer by layer, and thanks to the precise control of cell amount per spot, ratios between different cell types and the spot-to-spot spacing were evaluated by Pflaum et al. [41]. This technology would provide the possibility to create precisely designed microenvironments for cell culture, and therefore, due to the mimicking reached with this technique, a more realistic study of cell behavior could be performed.

Cell–ECM interactions, chemical signaling between cells, and direct cell–cell interactions are cell stimuli sources that cannot be understood as independent factors. It is known that cell–ECM interactions affects cell–cell tension, and therefore, cell–cell contact, resulting in an interdependence of cell–cell and cell–ECM interactions [42]. Recently, a microdevice of capable identifying the optimal combination of topographical (physical) and biochemical cues has been developed [43]. It was found that cells respond to topography and physical cues and that different biochemical signals alter cell response to topography.

Furthermore, a microtechnology based on polyacrilamide gels, capable of describing the effects of the combination of topographical, mechanical, chemical and intracellular electrical stimuli on a co-culture of fibroblasts and skeletal muscle cells, was described by Ricotti and co-workers [44]. The work demonstrated that muscle cell differentiation was synergistically enhanced with the combination of intracellular stimulation produced by piezoelectric nanostructures activated by ultrasounds. Cytokines were over-expressed by fibroblasts in the co-culture in correspondence to surface microtopography stimuli.

### 2.4. Fluidic Microenvironment

Fluidics and flow factors are vital for controlling the cellular microenvironment and cell behavior, as not only the diffusion and uptake of soluble factors depend on it, but it also affects physically the cells. Different microfluidic scenarios can be found in literature for cell analysis, that vary in configurations depending on the type of the studied cell or the application of the integrated assays. Moreover, specific shear and flow profiles are required in order to imitate in vivo conditions of cells, e.g., in blood vessels [45], and to be able to control those in microfluidics is very important. In certain occasions, when properties of drugs are tested, the flows are driven by peristaltic pumps that work at back pressures and generate pulses inside the microfluidic bioreactortors [46]. This could be a drawback in applications such as cell proliferation that require highly stable and continuous flow rates to generate constant shear stress on cells [47]. On the other hand, microfluidic devices can be used to manipulate the characteristics of cell matrixes. While some systems have been designed to simulate the mass transfer by convective transport of nutrients in capillary vessels in order to mimic the in vivo tissue environment, others use cell chambers that are isolated from the main fluid channel so that cells receive the nutrient via diffusive transfer [48].

**Figure 6:**
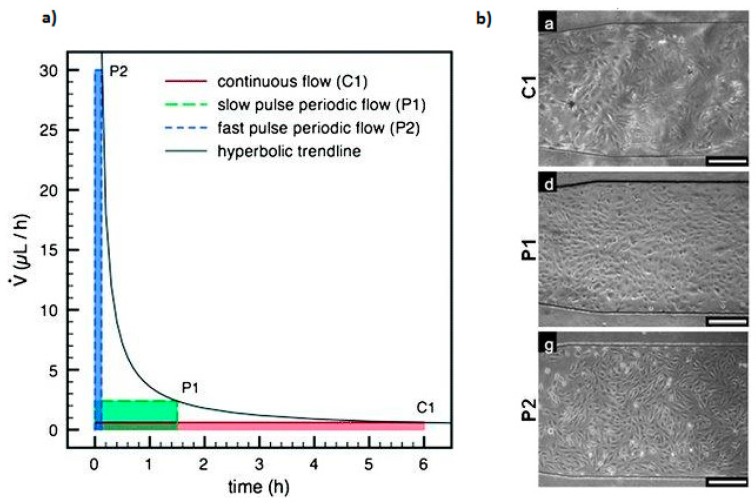
Study of different medium delivery strategies on long-term cell culture, varying the flow rate and the perfusion interval. (**a**) The same amount of medium was delivered using different perfusion conditions, assuming 6-h cycles. (**b**) The results for microfluidic C2C12 cell culture after four days show that a continuous perfusion with a stable flow condition leads to a heterogeneous growth (C1), while a fast pulse perfusion with periodic flow condition renders a uniform cell growth along the channel. White bars are 250 µm. Adapted from [49] with permission of The Royal Society of Chemistry.

Stefano et al. analyzed the effect of different flow conditions, from continuous to periodic perfusion, on long-term cell culture using microfluidic platforms for cell medium delivery [49]. The different perfusion conditions employed for C2C12 cell culture and the results obtained for each flow profiles are shown in Figure 6. On the one hand, the experiments demonstrated that a periodic medium delivery with fast pulses (P2) resulted in a homogeneous cell culture in terms of cell viability, colony morphology and maintenance of pluripotency markers. On the contrary, a continuous flow (C1) resulted in cell heterogeneity, with abnormal morphology and vesiculation.

Overall, continuous or pulsed medium delivery was used to maintain the cell microenvironments, but the optimal strategy for medium delivery is still an open issue. In this line, computational fluid dynamics is becoming a useful tool to try to understand the implications of fluid flow and nutrient transport on cell function and responses [48].

As mentioned above, flow control in microfluidics usually requires the use of external pumps or pneumatic fluid handling systems to generate continuous and stable or periodic and pulsed flows. Therefore, many investigations are being carried out to adequate fluid manipulation to the requirements of these novel microfluidic platforms. It is important to mention that many of these flow control components generate flow pneumatically by using air to pressurize the medium, therefore in case where the media which contains bicarbonate buffers, special attention must be paid to in the composition of the air used with correct percentage of CO_2_ and O_2_, to avoid pH changes in the medium. Therefore, it is recommendable to consider from the very beginning an adequate flow method [1].

Microfluidics also offers potential for tight medium composition control and devices that implement chemical gradients and droplet-based microfluidics are examples of widely used microfluidic systems that improve the sensitivity and complexity of experiments for studying cellular responses. Microfluidic devices involving gradient of soluble factors have been used to obtain a good differentiation of Stem Cells [50]. In order to avoid the exposure of cells to shear stress, a membrane-based microfluidic device has been developed by Kawada et al. to maintain, long term, an adequate delivery of soluble factors [51]. Chemical gradient devices can be driven under perfusion or statically: in continuous perfusion, the mixing of chemical species between two streams of fluid in laminar flow occurs due to diffusion, generating gradients of species at stream interfaces, and exposing the cells to these gradients across the microchannels. The perfusion also allows continuous nutrient supply and waste of removal, agreeing the in vivo microenvironment quality of most mammalian cell types. Limitations of perfusion include requirement of highly stable fluid flow and therefore fluid handling setups, relatively large volumes of reagents to maintain continuous and stable flowing gradients, and in situations where cell–cell communication is important perfusion is unsuitable for probing cellular responses. Instead, for the last case, static methods can be used, since static fluid preserves paracrine signaling. Static chemical gradients are formed between a source and a sink along a thin channel, and the gradient profile can be controlled by adjusting the input concentration, distance from source sink, or by changing the geometry of the channel. However, for this kind of device, special attention must be taken to avoid evaporation of the liquids, which could impair appropriate performance of the device increasing the concentrations, and the time required to set up the gradients is often rather long [52].

## 3. Large Arrays of Individually Addressable Cellular Systems under Controlled Microenvironment

To understand what is happening inside cells, it is necessary to perform biochemical analysis of cell components, and detect substances released by the cells. In general, for these type of analyses, it is necessary to create homogeneous populations of cells from a tissue, and to culture them to obtain enough material to be analyzed. Techniques like Fluorescently Activated Cell Sorting (FACS) enable the preparation of uniform populations of cells which can then be expanded in culture plates to produce large amounts of material to analyze.

Ideally, researchers would have some type of tool that would extract reliable information from small amount of cells, thus avoiding the need of cell culture. Such a tool could simultaneously also provide information about the nature of cells, avoiding in this case the need to separate different cell types within the tissue. The development of several microtechnologies enable the discrete manipulation and observation of small cellular systems, where each system may be comprised of a small number of cells, ranging from a single cell to several cells. Hence it is possible to observe and analyze a heterogeneous population of cells simultaneously, without the need of previous cell sorting or cell culture for expansion.

Currently, the most used cell culture methodologies rely on general protocols and large culture plates, only exceptionally taking into consideration substrate characteristic. As new discoveries have been made in how cells interact with their extracellular matrix and with each other, it has arisen the need to explore and study new ways to perfectly control and adapt the medium and the substrate to each type of cell [53]. For example, interesting cells such as stem cells or iPSCs usually requires specific characteristic for their culture, growth and differentiation that cannot always be achieved by conventional methods, thus requiring new techniques for cell culture [54]. In addition, creation of complex cultures such as body-on-a-chip requires new technologies that enable the recreation of physiological functions [55]. All this denotes the advantages and the need to control the cell microenvironment as much as possible to perform meaningful analytical measurements of the biology of cells.

Rapid screening methods and large samples have the potential to impact the development of predicting models for both conventional cell studies and the new models previously cited. The combination of high throughput methodologies with cell microenvironment control will pave the way to obtain high resolution information on dynamic cell behavior. Several methodological approaches and platforms have been developed to achieve large and dense arrays of individually addressable cellular systems to facilitate their study in a HTP manner. Those cellular systems, which can be fabricated in order to replicate the desired microenvironment, may vary in size ranging from individual single cells to groups of specific number of cells. The use of HTP methods enables parallelization and automation in both the creation of different microenvironments and the rapid screening of cellular outputs.

**Figure 7:**
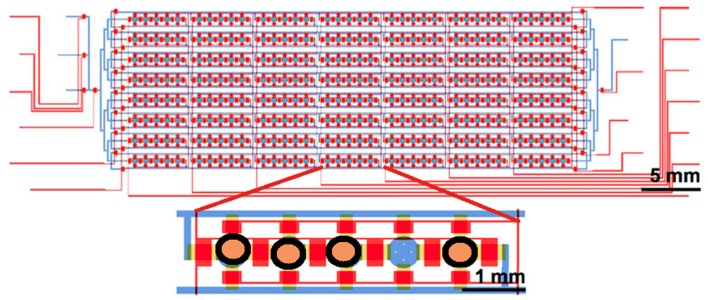
Schematic overview of a microfluidic large integration system where a great amount of single cells or cluster of cells can get entrapped simultaneously controlled by a valve system, adapted from [62] with the permission of Nature Publishing Group.

Microfluidic large-scale integration (mLSI) systems refer to microfluidic chips with large arrays of thousands of interconnected individually addressable microchambers [56], where individual cells can be independently isolated, assayed and recovered. mLSI, shown in Figure 7, are based on the integration of micromechanical elastomeric valves into multilayer polymeric microfluidic networks [57,58]. Recent applications of these type of platforms include single cell genome sequencing [59], single cell microRNA expression profiling [60], RT-qPCR, on more than 3000 cells [61], and HTP mammalian cell transfection and culturing [62].

**Figure 8:**
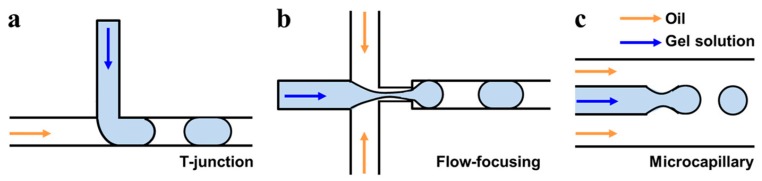
Three principle microfluidic geometries available for hydrogel droplet generation: (**a**) T-junction, (**b**) flow focusing, and (**c**) microcapillary. Reproduced from [70] with the permission of The Royal Society of Chemistry.

Droplet microfluidics is a type of microfluidic platform amenable for HTP analysis. Unlike continuous flow systems, droplet-based systems focus on creating discrete volumes with the use of immiscible phases. Droplets are usually generated by pressure-driven flow and are surrounded by an immiscible oil phase such that each droplet behaves as an individual microreactor or cell container [63] (Figure 8). Pressurized immiscible fluids are mixed at an orthogonal junction of two or more microchannels, combining cross-flow and viscous shear to generate monodisperse water-oil droplets with picolitre-scale volumes. Cells can be entrapped inside the droplets and reagents can be administered using very low volumes, even 1000 times smaller than the ones used in conventional microplate assays [64,65], enabling the creation of thousands of microenvironments within the same assay. Biocompatible surfactants for the development of the droplets and storage systems made of polymers are needed for the maintenance of cells in a liquid microenvironment in which they can proliferate and be controlled and manipulated. An application of this technology is the compartmentalization of single cells in droplets to enable the analysis of molecules that have been secreted by cells, such as antibodies secreted from single mouse hybridoma cells [66]. This process overcomes one of the major limitations of traditional flow cytometry and fluorescence-activated cell sorting techniques: the detection of secreted molecules. Full droplet microfluidics systems based on continuous workflow have also been used for manipulating and monitor continuous growth of bacteria populations. Bacteria populations are encapsulated on hundreds of microdroplets acting each as independent chemostats, microdroplets with specific chemical composition for continuous culture of microorganisms. The system allows studying the dynamics of bacterial populations in microdroplet chemostats as well as cellular responses to a range of stable or changing antibiotic concentrations [66,67]. DropSeq, is a recently developed HTP technology for single cell DNA sequencing based on droplet microfluidics. Individual cells are co-encapsulated with coded DNA-functionalized polymeric beads. It has been used for highly parallel genome-wide expression profiling of individual cells using nanoliter droplets [68]. Other droplet based methods have been developed, combining droplet microfluidics and reconfigurable flow-routing capabilities of integrated microvalve technology. It has been applied to multi-parameter analysis of single microbes and microbial communities [69].

Droplet-based techniques can be used to encapsulate the cells with different mediums at different concentrations and an enhanced mixing can be achieved due to internal recirculation. Furthermore, it allows highly parallelized experiments for HTP as described above [71]. This has also gained importance in the co-culture of cells since utilizing a microfluidic device in which co-encapsulation of cells was performed resulted in a technology capable of modulating signaling of microenvironment [72]. It is important to assure a correct performance of the droplet-microfluidics device to generate highly stable flows, and thus the control and stability of the flow rate. This is extremely important in order to avoid oscillations of pressure inside the system that could break its steady state and impede obtaining homogeneous and reproducible droplet size.

**Figure 9:**
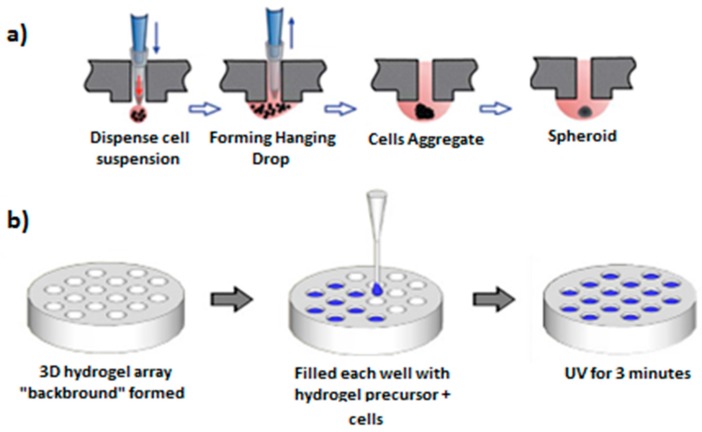
Fabrication of 3D cultures: (**a**) The suspension of cell in a hanging drop and the later formation of a spheroid containing all cells, adapted from [79] with permission of The Royal Society of Chemistry; and (**b**) the fabrication of a 3D cellular array by the filling of a hydrogel background with a solution of cells in a hydrogel precursor followed by a UV irradiation, adapted from [80] with permission of Elsevier.

Other strategies for the fabrication of highly dense cell arrays for HTP analysis include the fabrication of three-dimensional cell microarrays (Figure 9), for example on glass slides using robotic spotters, capable of dispensing and immobilizing nanolitters of material [73,74,75,76,77]. Suspensions of cells in different solutions containing proteins and other biomaterials are spotted on top of solid glass slides to create arrays of thousands of spatially addressable spots, each spot containing tens of cells. These types of arrays have been used for example for HTP toxicology assays [78], containing array spots of an average of 60 cells in each spot. A limitation of the most extended spotting robots is that the minimum spot size or drop volume is much larger than a single cell. Additionally, Popova et al. described a droplet-array (DA) sandwiching technology, a miniaturized platform for cell-based HTP assays, based on superhydrophobic–superhydrophilic micropatterns on nanoporous polymer films [75]. HTP 3D spheroid culture and drug testing using 384 hanging drops have also been reported [77,79].

**Figure 10:**
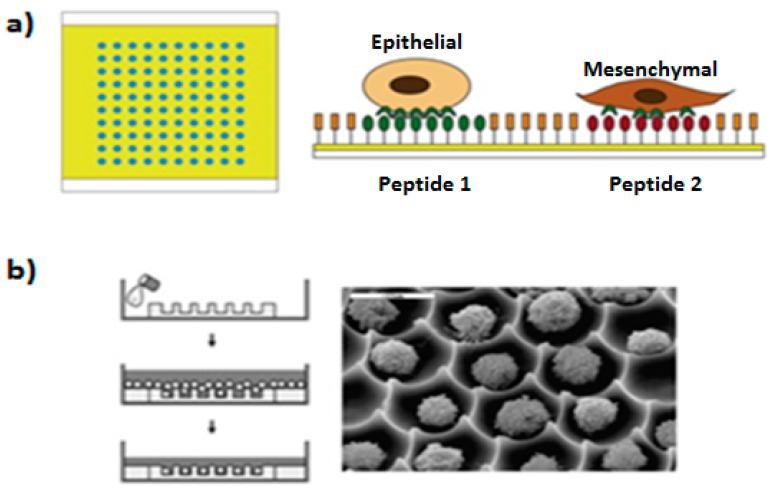
Non 3D cell culture formation: (**a**) The fabrication of cellular arrays in protein patterned substrate, where single cell or cluster of cells from different types can be isolated on top of the substrate specifically depending on their affinity to the patterned proteins, adapted from [83] with permission of Elsevier; and (**b**) the fabrication of cell arrays using craved microwells in a polymeric substrate where single cell or cluster of cells can be isolated in each well, adapted from [87] with permission of Elsevier.

When 3D culture is not required, other techniques enable cell adhesion to a substrate in a controlled manner (Figure 10). Those strategies include the fabrication of patterned substrates with thousands of individually addressable adhesive areas where individual cells or groups of cells may be immobilized. Adhesive patterned substrates may be done using low volume spotters such as Nanoenabler^®^ [81] and Nanoink^®^ (based on spotting and dip-pen nanolithography, respectively), or by microcontact printing [82]. Microcontact printing is a faster process for the creation of thousand of adhesive areas, because it only requires placing the substrate to pattern in contact with an inked stamp, for several minutes. Spotting instruments such as Nanoenabler or Nanoink, have the advantage of offering the possibility of creating multicomponent patterns. However, the time required for creating large arrays of adhesive areas is much higher than by microcontact printing because they rely on spotting on each of the areas to functionalize. Lin et al. reported the fabrication of printed arrays using spotting of multiple specific peptide ligands. They used a DNA microarrayers to demonstrate their utility in monitoring the induction of epithelial to mesenchymal transition (EMT) in murine mammary epithelial cells [83]. Recently, recombinant antibodies have been used as specific ligands for the formation of cell microarrays [84].

Finally, photolithography, and the fabrication of arrays of microwells or microsieves by die-cast molding of microstructures also enable the creation of highly dense cell arrays containing thousands of cellular systems, each of them trapped in microsieves or microwells [85]. Cellular systems created with these methods are comprised of one or several cells [17,86].

Techniques to create large density arrays of cellular systems are summarized in Table 1.

## 4. Monitoring

Besides high parallelization and microenvironment control, the other essential tool to progress on HTP cell studies is data acquisition and analysis. Important aspects related to different methods for data acquisition are: (1) provide real time measurements; (2) the need of stopping the cell culture media flow before measuring; (3) the use of label free method; or (4) the need to add extra reagents to obtain the data. All those aspects will influence the quality and frequency of data acquired. Higher frequency data production means more complex data analysis, but, at the same time, also provides a more accurate picture of the real situation.

Conventional methods such as immunofluorescence and immunohistochemistry using commercial fluorescence kits and microscopy observation are the leading technique for cell monitoring. While these techniques have been extremely useful for data acquisition from cellular behavior, most methods rely on costly-labeled substances that in some cases do not offer the required sensibility for cellular study. Apart from that, most of the current cell observation methods can only be used at the end of the assay, disabling the possibility of a real time observation.

**Figure 11:**
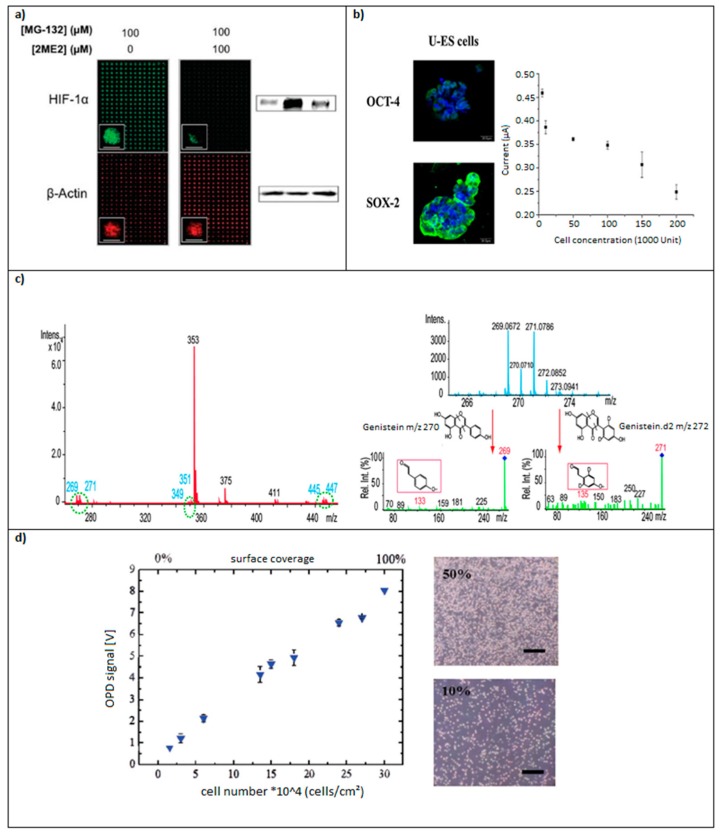
Selected examples of cell monitoring methods: (**a**) the conventional immunofluorescent imaging and Western blotting of proteins (hypoxia-inducible factor and actin) for cell characterization on cellular arrays, adapted with the permission of [73], copyright 2008 American Chemical Society; (**b**) the identification of undifferentiated mouse stem cells by the fluorescent marking of compounds only present in undifferentiated mouse stem cells, such as OCT-4 (octamer-binding transcription factor 4) and SOX-2 (Sex determining Region Y-box 2) and by the monitoring of the decrement of the electrochemical potential of the cellular membrane as the concentration of 1-Naphtyl phosphate (1-NP, not present in differentiated cells), adapted from [95] with permission from Elsevier; (**c**) mass spectra of extracts from MCF-7 cells incubated with 10 μM genistein and 10 μM genistein-d_2_ in DMEM on a microfluidic chip for 30 h, adapted with the permission of [98], copyright 2012 Amercian Chemical Society; and (**d**) light scattering signals measured in organic photodiodes following injection of increasing concentrations of suspension cells (Jurkat), adapted from [74] with permission of The Royal Society of Chemistry.

Nowadays, the development of new techniques for cell monitoring and observation is focused on label-free methodologies, which consist in the direct observation and characterization of cells without the need of secondary markers. The absence of secondary substances for the cell characterization allows lower cost and more efficient cellular studies. These techniques not only retain the integrity of the cell culture and their biomolecules, but also allow to some extent the observation and monitoring of real-time events (Figure 11). These techniques also provide a higher sensibility up to a certain degree, allowing the monitoring of several individual events in a HTP manner [88,89,90].

Following the route of label-free techniques, new integrated sensors have been applied within microfluidic devices for biological applications and therefore increase the existing tool box of monitoring methods. Conventionally, light sources use laser or lamps; several alternatives were developed, such as the incorporation of light self-emitting diodes (LEDs) and organic photodiodes. Other researchers integrated optical sensors into the microfluidics devices, using light scattering, absorption, transmission and fluorescence [74,91]. For a more real-time like cell analysis, high-speed cameras have also been applied in order to obtain as much information from the sources and the sensors in the lowest time lapse [92]. A new approach was investigated by Casterllanau et al. which involved the integration of particles working as sensors into the device. These fluorescent particles were used for the identification of both single-cells and small collection of cells [91]. Newer nanorobotic manipulation inside electron scanning based microscopes has also been postulated as new ways to characterize cell cultures [93].

Physicochemical factors and biophysicials processes have also been used as novel techniques for the cellular characterization and monitoring of cells. Electro-analytical methods such as voltammetry, potentiometry and impedance spectroscopy were also employed. They were used to study the electrical properties of cell cultures, as well as electrical-dependable cell processes, such as viability, proliferation and motility changes. Impedance spectroscopy was used along with light scattering for cell analysis. In addition, an electro-analytical method based on trans-epithelial electrical resistance was used for the analysis of physiological barriers [94,95].

Novel magnetic based monitoring techniques were developed considering the magnetic properties of cells, especially during the up-take and transfer. In particular, magnetic nanoparticles were proposed as excellent tools for both cell separation and detection techniques. Integrated magnetic biosensors with superparamagnetic nanoparticles were functionalized to identify biological species and to quantify the released products [96,97].

Finally, mass spectroscopy was postulated as a powerful tool for qualitative and quantitative analysis of metabolites of cells. Mass spectroscopy has been widely used for the detection of secreted substances by cells in batch experiments. Nowadays, new microchips have been created for the simultaneous culture of cells and the detection in situ of some of the secreted substances by mass spectroscopy [98].

## 5. Conclusions

There is a high demand and a constant increase in the development of microtechnologies for cell analysis. During the last two decades, we have witnessed a number of key developments in the area of microtechnology and their applications in cell culture and analysis. For example, the manipulation of the topography of substrates creating micro- and nano-patterns has allowed evaluating the effect of topography in cell culture and differentiation; the creation of chemical patterns with precision in the microscale has enabled to look at the importance of cell adhesion, cell shape and cell–cell contact in their behavior; controlling the composition of cell culture medium, through microfluidics networks helped understanding the effect of the nutrients concentration on cell growth; and novel printing and dispensing techniques, in combination with biomaterials, enabled to create micro-scaled 3D cell cultures allowing to look at cellular systems that resemble tissue architectures. All these recently developed tools enable performing experiments with a higher level of control in the cellular microenvironment than those classically conducted on flat cell culture plates.

Additionally, a number of strategies have been developed for the fabrication of dense arrays of cellular systems, where a large number of systems are created for subsequent stimulation, observation and monitoring. Depending on the methodology, the cellular systems can be comprised of a single cell or few cells; each of those system may be anchored to a surface or suspended in a fluid, and they may be distributed in a plane or in a 3D distribution, for example embedded into a gel structure. The possibility of creating a collection of individually addressable cellular systems improves both, the statistical analysis of the properties of cells, and the possibility to observe heterogeneous behavior on a cell population.

Finally, there is also an increasing interest in monitoring methods for cell characterization. Newcomers in the area of monitoring cells include integrated optical and electrical sensors and label-free characterization techniques.

The usages of microtechnologies in cell biology will not only provide the possibility of acquiring new data, but it will also allow to perform analysis using small amount of biological material. Microtechnologies, such as microfluidics, is opening the possibility of undergoing subsequent analysis on the same cellular system, reducing in this way the operation time, the need of cell separation, and the need of cell expansion. Depending on the aim of the study, each different methodology offers a set of benefits and disadvantages that could influence the election of one technique over another.

The transition from macroscale to microscale in cell analysis is still developing, and it will be only through the integration of several microtechnologies that we will obtain a new generation of cell analysis microsystems able to provide information on those still undiscovered steps of cell function and differentiation. In the future, highly integrated microfluidic analytical devices will find applications in different areas, such as drug discovery and cell studies for regenerative medicine. These microfluidic platforms must enable cell culture under controlled microenvironment and lead to a possible HTP and real time analysis. For this purpose, it will be necessary the integration of a variable number of newly developed micro- and nano-technologies, that will allow controlling the topography, the surface chemistry, soluble factors, mechanical forces and cell–cell contacts; and technology for monitoring cell phenotype and genotype with high spatial and temporal resolution on a HTP manner.

## Figures and Tables

**Table 1:** Comparison of the different novel techniques for creation of cellular cultures.

Technique	Definition	Cell Isolation Capacity	2D/3D Culture	Other Characteristics
**Microfluidic large-scale integration systems (mLSI)**	Interconnected addressable microchambers for isolation and recovering of cells	Multiple and single cell	Two-dimensional cultures	Thousands of addressable events
**Droplet microfluidic platforms**	Droplet-based systems for cell entrapping in discrete volumes of immiscible phases	Multiple and single cell	Two- and three-dimensional cultures	Thousands of addressable events Low volume requirement
**3D microfluidic platforms**	Spotting of biological suspension on addressable spots	Multiple cells	Three-dimensional cultures	Low volume requirement
**Hanging drops platforms**	Entrapping of spheroid cultures inside a hanging drop	Multiple cells	Three-dimensional cultures	Hundreds of addressable events
**Patterned substrates**	Patterning of chemical adhesive areas for cell immobilization	Multiple and single cell	Two-dimensional cultures	Thousands of addressable events Low volume requirement Fast processes
**Microwell structures**	Molding of microsieves or microwells for cellular trapping	Multiple and single cell	Two-dimensional cultures	Thousands of addressable events
